# The Treatment of Post-Partum Hæmorrhage

**Published:** 1901-05-11

**Authors:** 


### Editor's Letter-Box.

### The Treatment of Post-Partum Hæmorrhage.

To the Editor of *The Hospital*.

Mr. Stanmore Bishop, referring to various criticisms made on his proposal to control post-partum hæmorrhage by pressure upon the aorta, writes:—

First: the body of the vertebra against which the aorta has to be compressed is roughly convex, and the aorta does not lie on the front of this surface but somewhat to the left, so that the direction of the pressure by which the aorta will be controlled tends to press the vena cava out of the way—should it reach that vessel at all, but the chances are but very small even of this.

Second: as to the possibility of feeling the aorta in a very adipose person. If one looks at a representation of a frozen section, such as the plate in "Kelly's Operative Gynæcology," it is astonishing what a large amount of the sectional area of the body in this region is occupied by the spine and spinal muscles, and how near the anterior surface of the spine is to the abdominal wall. It seems to me that those who raise this objection are unconsciously thinking of a fat woman at an ordinary time, with well braced-up abdominal muscles, not of the woman as she is just after labour, with a sudden removal of a mass which has slowly distended, stretched, and weakened in tone all her muscular and fascial wall. This wall now lies limp and loose over a space which can only contain intestine which for months has been packed away under the ribs or in the renal regions, and does not all at once fill the available space. Besides this intestine there is the collapsed uterus, containing more or less blood, but no firmly resistant solid, and easily pushed on one side.

A thick cushion of fat well supported by firm elastic muscles, backed by the elastic and distended intestine, is a very different thing to feel through than the same or even a greater amount of fat, with no backing at all worth mention, lying loosely over an empty potential cavity. It is necessary to keep this essential alteration clearly in mind.

Taking these two last points together, you will see that this is really only a theoretical difficulty, and in practice I can answer for it you will find it non-existent.

